# v-Src Causes Chromosome Bridges in a Caffeine-Sensitive Manner by Generating DNA Damage

**DOI:** 10.3390/ijms17060871

**Published:** 2016-06-02

**Authors:** Masayoshi Ikeuchi, Yasunori Fukumoto, Takuya Honda, Takahisa Kuga, Youhei Saito, Naoto Yamaguchi, Yuji Nakayama

**Affiliations:** 1 Department of Biochemistry & Molecular Biology, Kyoto Pharmaceutical University, Kyoto 607-8414, Japan; ky11020@poppy.kyoto-phu.ac.jp (M.I.); kuga@mb.kyoto-phu.ac.jp (T.K.); ysaito@mb.kyoto-phu.ac.jp (Y.S.); 2 Department of Molecular Cell Biology, Graduate School of Pharmaceutical Sciences, Chiba University, Chiba 260-8675, Japan; fukumoto@faculty.chiba-u.jp (Y.F.); t-honda@chiba-u.jp (T.H.); nyama@faculty.chiba-u.jp (N.Y.)

**Keywords:** v-Src, chromosome bridge, DNA damage, caffeine

## Abstract

An increase in Src activity is commonly observed in epithelial cancers. Aberrant activation of the kinase activity is associated with malignant progression. However, the mechanisms that underlie the Src-induced malignant progression of cancer are not completely understood. We show here that v-Src, an oncogene that was first identified from a Rous sarcoma virus and a mutant variant of c-Src, leads to an increase in the number of anaphase and telophase cells having chromosome bridges. v-Src increases the number of γH2AX foci, and this increase is inhibited by treatment with PP2, a Src kinase inhibitor. v-Src induces the phosphorylation of KAP1 at Ser824, Chk2 at Thr68, and Chk1 at Ser345, suggesting the activation of the ATM/ATR pathway. Caffeine decreases the number of cells having chromosome bridges at a concentration incapable of inhibiting Chk1 phosphorylation at Ser345. These results suggest that v-Src induces chromosome bridges via generation of DNA damage and the subsequent DNA damage response, possibly by homologous recombination. A chromosome bridge gives rise to the accumulation of DNA damage directly through chromosome breakage and indirectly through cytokinesis failure-induced multinucleation. We propose that v-Src-induced chromosome bridge formation is one of the causes of the v-Src-induced malignant progression of cancer cells.

## 1. Introduction

Protein-tyrosine kinases can be classified into two groups: receptor-type tyrosine kinases and non-receptor-type tyrosine kinases. c-Src, a non-receptor type protein tyrosine kinase, plays a role in a variety of cellular process, including cytoskeletal reorganization, migration, and proliferation [1]. The activity of c-Src is regulated by Csk-catalyzed phosphorylation of the C-terminal tyrosine residue. This phosphorylation generates intramolecular binding between the SH2 domain and the phosphorylated tyrosine residue, resulting in the formation of closed conformation and inhibition of the kinase activity. Displacement of this intramolecular binding by a higher affinity ligand prevents c-Src from forming the closed conformation and activates the kinase activity of c-Src.

v-Src is an oncogene that was first identified from the Rous sarcoma virus [2]. v-Src is a mutant variant of the cellular proto-oncogene c-Src, due to the loss of the tyrosine residue typically found in c-Src at the C-terminus; therefore, v-Src can escape from Csk and is highly activated without binding to the ligands, contributing to the oncogenic character of v-Src. Upon v-Src expression, cells lose actin stress fibers and focal-adhesion, cell–cell interactions are disrupted, and anoikis resistance is gained. An increase in Src activity is commonly observed in epithelial cancers [3] and is attributed to the mutation of Src and an elevation of Src expression [3]. Similar to v-Src, the aberrantly activated Src can evoke various cellular processes and cause extensive changes in gene expression, leading to a malignant progression. However, the mechanisms that underlie Src-induced malignant progression are not completely understood.

We and other researchers have previously reported that Src family kinases participate in the regulation of cell division [4,5,6,7,8,9,10]. Given that cell division is tightly regulated to prevent asymmetrical segregation of replicated DNA, aberrantly activated Src would result in abnormal cell division. Recently, we reported that v-Src induces failure of cytokinesis through delocalization of the mitotic kinase Aurora B [11]. Furthermore, we found that v-Src leads to an increase in the chromosome bridges in anaphase and telophase cells [11]. The next step is to uncover the mechanisms underlying the v-Src-induced chromosome bridge formation. A chromosome bridge gives rise to the accumulation of DNA damage directly through successive DNA breakage and indirectly through cytokinesis failure-induced multinucleation. Therefore, we propose that v-Src-induced chromosome bridges are one of the causes of the v-Src-induced malignant progression of cancer cells.

## 2. Results

**Figure 1:**
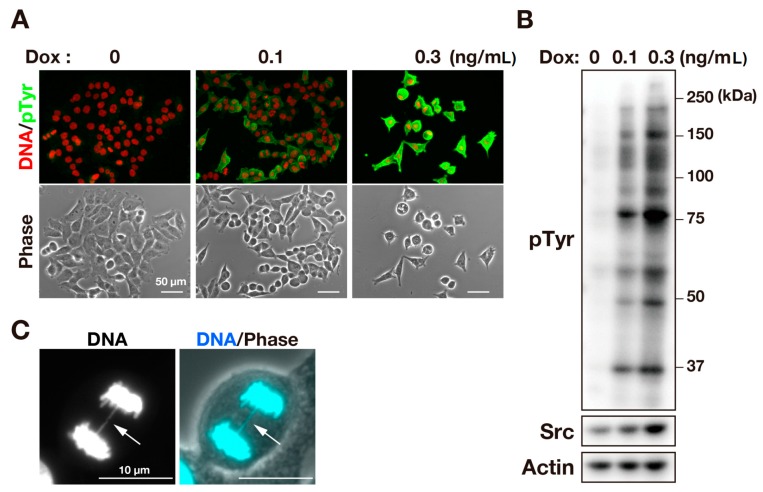
v-Src induces chromosome bridge formation. HCT116/v-Src cells were cultured with Dox at the indicated concentrations for three days. (**A**) The cells were fixed with PBS containing 4% formaldehyde for 20 min at room temperature and stained for pTyr (green) and DNA (red); (**B**) Whole cell lysates were subjected to a Western blot analysis. Blots were probed with anti-phosphotyrosine (pTyr), anti-Src (GD11), and anti-actin (loading control) antibodies; (**C**) The cells were fixed and stained for DNA (cyan). The arrow indicates a chromosome bridge.

To examine the effects of v-Src on chromosome segregation, we used an HCT116/v-Src cell line capable of a tetracycline-inducible expression of v-Src. Upon treatment with doxycycline (Dox), HCT116/v-Src cells expressed v-Src in a concentration-dependent manner, leading to an increase in the level of tyrosine phosphorylation of proteins (Figure 1A,B). Immunofluorescence microscopy showed that Dox treatment induced cell rounding. These cells exhibited an interphase nucleus, indicating that cell rounding was not caused by the mitotic entry (Figure 1A). An increase in the levels of tyrosine phosphorylation and cell rounding are characteristic phenotypes for v-Src expression [11]. When we scrutinized chromosome segregation, we observed chromosome bridges in anaphase and telophase cells. The number of cells containing chromosome bridges was significantly increased by the inducible expression of v-Src (Figure 1C, see also Figure 4A), as described previously [11]. These results confirm the effect of v-Src on chromosome segregation.

**Figure 2:**
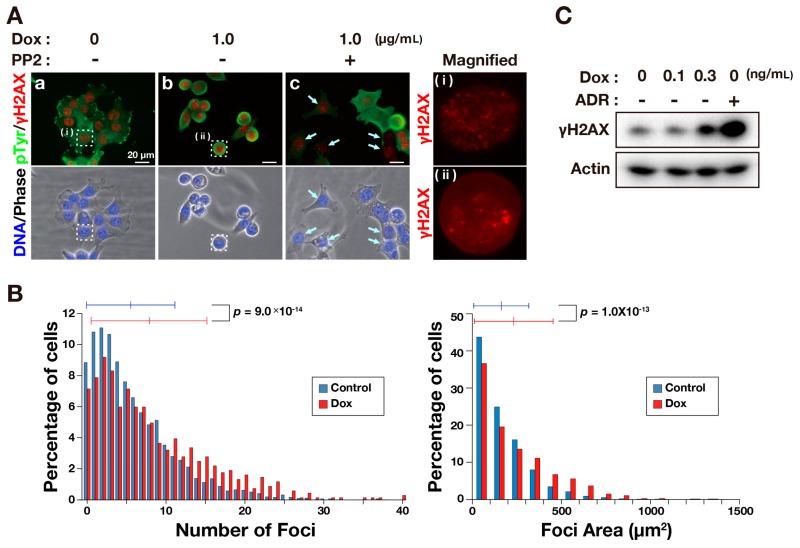

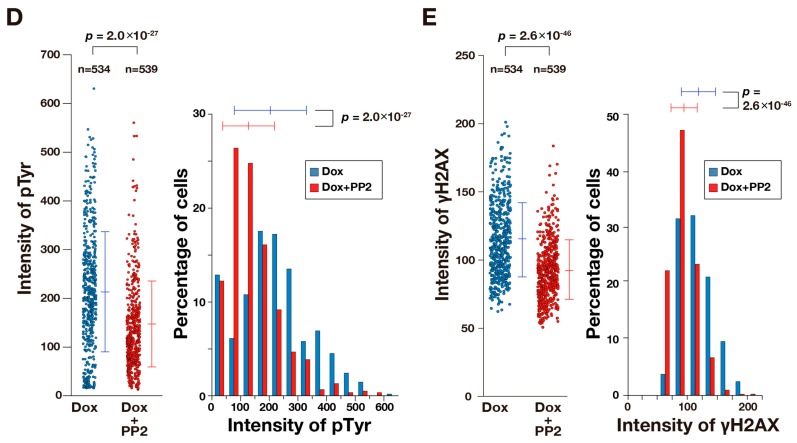
v-Src induces DNA damage. (**A**) HCT116/v-Src cells were cultured with 1.0 μg/mL Dox for 12 h in the presence or absence of 10 μM PP2. The cells were fixed with PBS containing 4% paraformaldehyde and 20% methanol for 20 min at room temperature and stained for pTyr (green), γH2AX (red), and DNA (blue). Arrows indicate cells showing lower levels of pTyr staining. The boxed regions are magnified in the right panels. Bars, 20 μm; (**B**) HCT116/v-Src cells were cultured with or without 1.0 μg/mL Dox for 12 h. The cells were fixed with PBS containing 20% methanol and 4% formaldehyde for 20 min at room temperature and stained for γH2AX and DNA. The fluorescence signals were analyzed by an image analyzer. The left histogram shows the number of γH2AX foci per nucleus. The right histogram shows the total area of γH2AX foci per nucleus. The results represent the mean ± S.D. None, *n* = 2870; 1.0 μg/mL Dox, *n* = 686; (**C**) Cells were cultured with Dox at the indicated concentrations for three days or 300 ng/mL Adriamycin (ADR) for one day as a positive control. Whole cell lysates were subjected to a Western blot analysis. Blots were probed with anti-γH2AX and anti-actin (loading control). A representative result of two independent experiments is shown; (**D**,**E**) Cells were cultured with 1.0 μg/mL Dox for 12 h in the absence or presence of 10 μM PP2. Then, the cells were fixed and stained as described in **A**. The fluorescence signals were analyzed by an image analyzer. In **D**, fluorescence intensities of pTyr in individual cells were plotted with the mean ± S.D. (*n* > 534) in the left, and data were converted to a histogram plot shown on the right; In **E**, fluorescence intensity of γH2AX in an individual nucleus was plotted with the mean ± S.D. (*n* > 534) on the left, and data were converted to a histogram plot as shown on the right. *p* values were calculated using a two-tailed Student’s *t*-test.

A chromosome bridge can result from DNA damage. Indeed, the number of chromosome bridge-containing cells increase with telomere dysfunction [12,13] and DNA double strand breaks following ionizing radiation [14]. To investigate whether the DNA damage was caused by v-Src, we stained cells for γH2AX as a marker of DNA damage. Immunofluorescence microscopy showed that a 12-h Dox treatment induced an increase in the levels of tyrosine phosphorylation together with cell rounding in interphase cells, indicating v-Src expression. Staining for γH2AX showed foci formation in the nuclei (Figure 2(Aa,Ab)), and was analyzed by a high-content automated imaging system. Histograms of the number of γH2AX foci (Figure 2B left) and total area of γH2AX foci (Figure 2B right) per nucleus were both shifted to the right by v-Src expression, indicating increases in the number of the γH2AX foci and total area of γH2AX foci upon v-Src expression. These increases in the γH2AX signal were also confirmed by Western blot analysis (Figure 2C). These results suggest that v-Src induces DNA damage.

We next used the Src selective inhibitor PP2 to examine whether DNA damage was associated with the kinase activity of v-Src. HCT116/v-Src cells were treated with Dox and with or without PP2, stained for phosphotyrosine and γH2AX (Figure 2(Ab,Ac)), and analyzed by a high-content automated imaging system. Fluorescence intensities of phosphotyrosine staining are shown in a dot plot (Figure 2D left) and a histogram (Figure 2D right). Both graphs show that a PP2 treatment reduced the level of tyrosine phosphorylation, suggesting an inhibition of v-Src kinase activity by PP2. Fluorescence intensities of γH2AX presented in the dot plot (Figure 2E left) and the histogram (Figure 2E right) show a decrease in the levels of γH2AX with a PP2 treatment. Furthermore, the cells that are indicated by arrows in Figure 2(Ac) exhibit lower levels of both γH2AX signals and phosphotyrosine signals. These results suggest that tyrosine phosphorylation catalyzed by v-Src is involved in v-Src-induced DNA damage.

**Figure 3:**
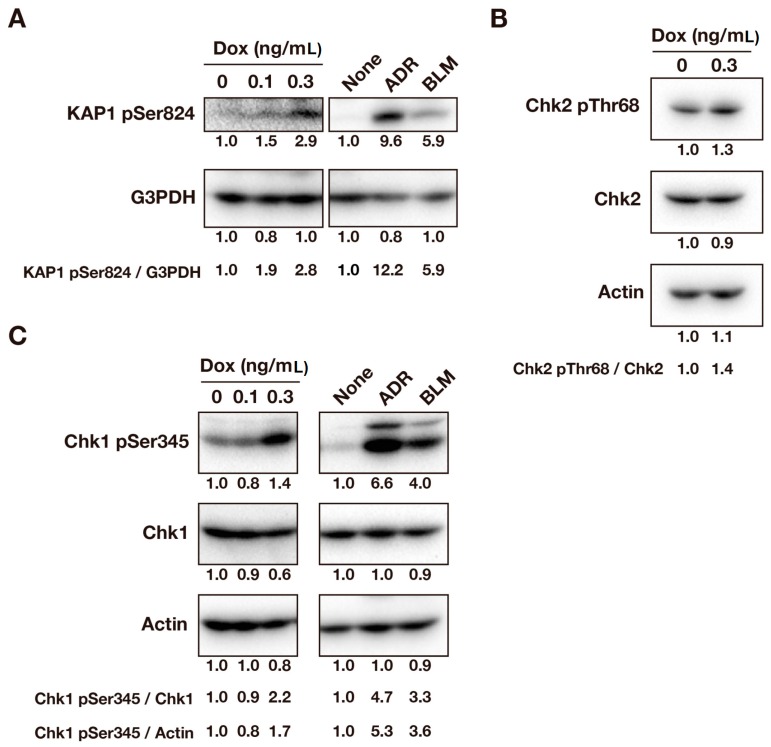
v-Src induces ATM/ATR activation. HCT116/v-Src cells were cultured with Dox at the indicated concentrations for three days. ADR (300 ng/mL) and BLM (4.46 μg/mL) were used as the positive control and cells were treated for one day before lysate preparation. Whole cell lysates were subjected to a Western blot analysis. A representative result of two independent experiments is shown. (**A**) Blots were probed with anti-KAP1 pSer824 and anti-G3PDH (loading control) antibodies. KAP1 phosphorylation at Ser824 was quantified by measuring the signal intensities of the bands. The ratios to the levels of G3PDH are shown; (**B**) Blots were probed with anti-Chk2 pThr68, anti-Chk2, and anti-actin (loading control) antibodies. Chk2 phosphorylation at Thr68 was quantified by measuring the signal intensities of the bands. The ratios to the Chk2 are shown; (**C**) Blots were probed with anti-Chk1 pSer345, anti-Chk1, and anti-actin (loading control) antibodies. Chk1 phosphorylation at Ser345 was quantified by measuring the signal intensities of the bands. The ratios to the Chk1 and actin levels are shown.

DNA damage elicits a broad range of cellular responses, such as cell cycle arrest, apoptosis, and DNA repair through the activation of the ATM/ATR pathway. ATR phosphorylates Chk1 at Ser345, and ATM phosphorylates Chk2 at Thr68 and KAP1 at Ser824 [15,16]. These phosphorylations were examined to confirm that v-Src caused DNA damage. As positive controls, phosphorylated bands of Chk1 and KAP1 were clearly observed upon treatment of cells with Adriamycin (ADR) and bleomycin (BLM) for one day before lysate preparation. HCT116/v-Src cells were treated with Dox for three days and subjected to a Western blot analysis. Treatment of cells with Dox increased phosphorylations of KAP1 at Ser824 (Figure 3A), Chk2 at Thr68 (Figure 3B), and Chk1 at Ser345 (Figure 3C). These results confirm that v-Src causes DNA damage and elicits activation of the ATM/ATR pathway.

**Figure 4:**
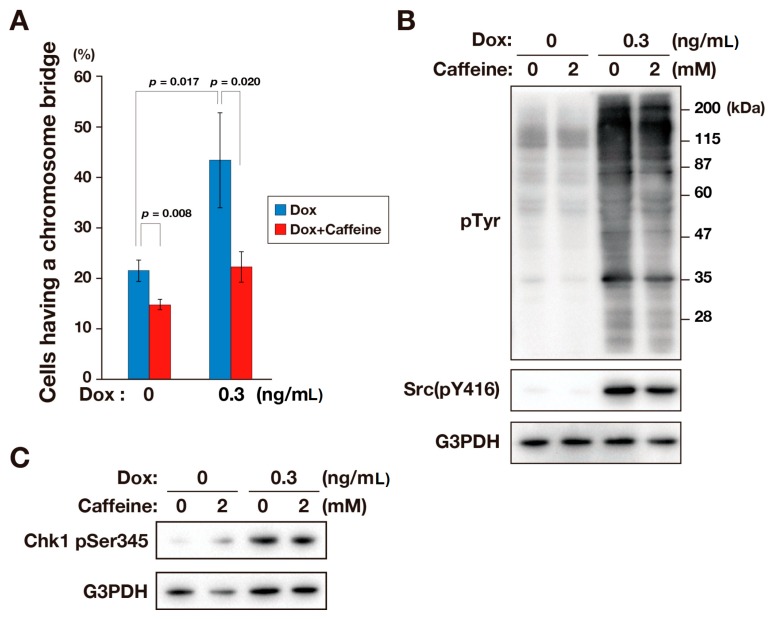
Caffeine inhibits the formation of chromosome bridges. HCT116/v-Src cells were cultured with Dox for three days at the indicated concentrations. The cells were treated with or without 2 mM caffeine for one day before fixation or lysate preparation. (**A**) The percentage of anaphase and telophase cells with a chromosome bridge were plotted. The results represent the mean ± S.D. from three independent experiments. *p* values were calculated using a two-tailed Student’s *t*-test; (**B**) Whole cell lysates were subjected to a Western blot analysis. Blots were probed with anti-phosphotyrosine (pTyr), anti-Src (pY416), and anti-G3PDH (loading control) antibodies; (**C**) Whole cell lysates were subjected to a Western blot analysis. Blots were probed with anti-Chk1 pSer345 and anti-G3PDH (loading control) antibodies. In **B** and **C**, a representative result of two independent experiments is shown.

Caffeine has been shown to inhibit DNA damage responses triggered by the ATM/ATR pathway [17,18,19,20,21]. To examine whether caffeine inhibited the v-Src-induced chromosome bridge formation, HCT116/v-Src cells were treated with 0.3 ng/mL Dox for three days together with caffeine for one day before fixation. The fixed cells were stained for DNA, and anaphase and telophase cells were examined for the presence of chromosome bridges. The number of cells having a chromosome bridge was increased by v-Src, and this increase was reduced in the presence of 2 mM caffeine (Figure 4A). A Western blot analysis using an anti-phosphotyrosine antibody showed no difference in the levels of phosphotyrosine between caffeine-treated and untreated cells, indicating that caffeine did not affect v-Src kinase activity (Figure 4B). As shown in Figure 3, phosphorylation of Chk1 at Ser345 was detected in cells treated with 0.3 ng/mL Dox, and this phosphorylation was not inhibited by 2 mM caffeine, suggesting that caffeine does not inhibit ATR kinase activity in the experimental conditions used here, consistent with previous studies [22,23]. These results suggest that caffeine inhibits v-Src-induced chromosome bridge formation without inhibiting ATR kinase.

## 3. Discussion

In the present study, we show that v-Src induces chromosome bridge formation via generation of DNA damages and the resultant DNA damage response. A chromosome bridge gives rise to a direct accumulation of DNA damage through chromosome breakage [24,25]. In addition, a chromosome bridge accidentally causes cytokinesis failure, inducing aneuploidy through multinucleation [13,26,27]. In short, v-Src may induce chromosome instability. The chromosome instability is a critical feature that can enable malignant progression through inactivation of tumor suppressor genes, activation of oncogenes, and amplification of drug resistance genes [28]. Our results provide the possible mechanisms that underlie the v-Src-induced malignant progression; v-Src-induced chromosome bridge formation may promote aneuploidy and thereby may be responsible for overwhelming v-Src-induced growth suppression.

The cell clone used in this study is capable of expressing v-Src upon Dox treatment. The expression levels of v-Src depend on the concentration of Dox added to the culture. This has a benefit for the investigation of v-Src, since v-Src expression at higher levels induces a variety of effects including detachment of cells from culture dish. However, v-Src that was induced by low concentration of Dox for a long time induces an increase in protein-tyrosine phosphorylation without detachment of cells. Therefore, when cells are examined for DNA damage induction in a short period, cells were treated with higher concentration of Dox at 1 μg/mL. On the contrary, when cells were examined for chromosome bridge formation for three days, cells were treated with lower concentration of Dox at 0.1–0.3 ng/mL.

Given that a chromosome bridge gives rise to a direct accumulation of DNA damage through chromosome breakage [24,25], DNA damage observed here would be caused by breakage of the chromosome bridges. To exclude this possibility, we examined whether DNA damage was induced in a shorter period of Dox treatment at 1 μg/mL. Considering that several hours would be required for accumulation of v-Src protein and an increase in tyrosine phosphorylation, DNA damage observed after 12-h Dox treatment is unlikely to be caused by the breakage of chromosome bridges. Therefore, we conclude that v-Src induces DNA damage.

v-Src-induced chromosome bridge formation but not Chk1 phosphorylation at Ser345 was inhibited by treating the cells with caffeine (Figure 3 and Figure 4A). This suggests that caffeine may block checkpoint responses without inhibiting the ATM/ATR pathway, consistent with previous studies [22,23]. It was reported that caffeine suppresses homologous recombination (HR) by interfering with RAD51-mediated joint molecule formation without inhibiting ATM and ATR kinases [23]. Acilan reported that nonhomologous end joining (NHEJ) helps prevent bridges, and HR leads to chromosome bridge formation in the absence of NHEJ [14]. These results suggest that HR may be involved in v-Src-induced chromosome bridge formation. Given that defects in DNA repair pathway leads to the accumulation of DNA damage, our findings can raise the possibility that v-Src would disrupt NHEJ and thereby leads to the accumulation of DNA damage, resulting in an induction of chromosome bridge formation in an HR-dependent manner. Further studies, including proteomics analyses of v-Src substrates, will uncover the mechanisms that underlie v-Src-induced DNA damage and chromosome bridge formation.

Cell division is tightly regulated to ensure a faithful inheritance of chromosomes, and protein phosphorylation of Ser/Thr residues plays an important role. Furthermore, we and other researchers have reported that Src family tyrosine kinases are involved in mitotic processes [4,5,6,7,8,9,10]. Since kinases are spatio-temporally regulated, it is likely that abnormally activated kinases disrupt the cell division process. Indeed, we found that v-Src induces binucleated cells through delocalization of the mitotic kinase Aurora B and the kinesin Mklp1 from the spindle midzone at the anaphase, thereby causing furrow regression [11]. Binucleated cells tend to cause abnormal cell division via the following cell cycle; v-Src leads to aneuploid cells by inducing cytokinesis failure.

Additionally, we demonstrated the induction of DNA damage by v-Src and the subsequent generation of chromosome bridges. A chromosome bridge activates the abscission checkpoint that delays abscission until the chromosome bridge is resolved [29,30,31]. In the presence of a chromosome bridge, ANCHR associates with VPS4, controls the abscission timing and prevents multinucleation [29,30,31]. Knockdown of ANCHR or inhibition of Aurora B kinase in chromosome bridge-containing cells allows abscission, resulting in furrow regression and multinucleation [29,30]. That is, the abscission checkpoint plays a role in avoiding cytokinesis failure in chromosome bridge-containing cells. We reported that v-Src delocalizes Aurora B from the anaphase midzone and midbody and induces the furrow regression [11]. Given that the abscission checkpoint requires Aurora B kinase activity, v-Src may induce the silencing of the abscission checkpoint via Aurora B delocalization, generating multinucleated cells via a furrow regression in chromosome bridge-containing cells. Taken together, v-Src may promote aneuploidy in chromosome bridge-containing cells through checkpoint silencing and subsequent cytokinesis failure.

Due to a loss of the c-terminal tyrosine residue, the kinase activity of v-Src is continuously elevated and v-Src induces diverse effects on many cellular processes [3]. Upon v-Src expression, a loss of actin stress fibers and focal-adhesion, a disruption of cell–cell interactions, and resistance to anoikis are evoked. However, the effect on cell proliferation is controversial. An enhancement of cell proliferation has been observed; stimulation of the MEK/ERK pathway [32] and G1/S cyclin-CDK complexes and suppression of the CDK inhibitor p27 [33,34] were reported. Conversely, the suppression of cell proliferation using *in vitro* and *in vivo* experiments has also been reported [3,35]. In addition, upon v-Src expression together with inhibition of Ras and PI3-kinase signaling, apoptosis was induced in Rat-2 fibroblast cells [36]. We also observed a suppression in cell proliferation in human cervix HeLa S3, colorectal HCT116, and mouse fibroblast NIH3T3 cells upon inducible v-Src expression [11]. These findings raise the possibility that v-Src-enhanced cell proliferation depends on the cell type, some of which require further changes that would be indirectly produced by v-Src for the enhancement of cell proliferation.

What kind of changes turn on the switch that unlocks v-Src-induced growth suppression? As described, v-Src may promote aneuploidy through chromosome bridge formation and cytokinesis failure; the genetic alteration may turn on the switch. When v-Src induces growth suppression, this suppression is supposed to last until mutations accumulate and overwhelm the suppression or until cell death. Therefore, v-Src-induced growth suppression may be a selective pressure for the accumulation of additional mutations that allow cells to proliferate by overwhelming the v-Src-induced growth suppression. Given that v-Src may promote aneuploidy, it is interesting to suppose that, in addition to its well-known canonical roles in cancer development, v-Src has at least two more additional roles in cancer development. These roles may include selective pressure by growth suppression and a driving force for mutations by chromosome bridge formation and cytokinesis failure. Further studies are necessary to fully describe the roles of v-Src in cancer development and malignant progression.

## 4. Materials and Methods

### 4.1. Cells and Cell Culture

HCT116 (human colon carcinoma) cells capable of inducing the expression of v-Src [11] were cultured in Dulbecco’s modified Eagle medium containing 5% fetal bovine serum with 20 mM Hepes-NaOH (pH 7.4). v-Src was inducibly expressed by incubation of these cells with doxycycline (Dox) at concentrations of 0.1 ng/mL–1 μg/mL, these details are shown in the figure legends.

### 4.2. Chemicals

Adriamycin (Sigma-Aldrich, St. Louis, MO, USA) and bleomycin (Nihonkayaku, Tokyo, Japan) were used to induce DNA damage at concentrations of 300 ng/mL and 4.46 μg/mL (3.0 μM), respectively. Caffeine (Wako, Osaka, Japan) was used at 2 mM for 24 h. These chemicals were dissolved in distilled water as stock solutions. PP2 (Calbiochem, San Diego, CA, USA), a selective inhibitor for the Src family kinase, was dissolved in dimethyl sulfoxide and used at 10 μM.

### 4.3. Antibodies

We used rabbit monoclonal anti-phospho-histone H2A.X (Ser139) (γH2AX) (1:400; 2577S, Cell Signaling Technology, Danvers, MA, USA) and mouse monoclonal anti-phosphotyrosine (1:400; clone 4G10, Merck Millipore, Darmstadt, Germany) primary antibodies for immunofluorescence analyses. We also used Alexa Fluor 488-, Alexa Fluor546-, or Alexa Fluor 555-labeled donkey anti-mouse IgG and donkey anti-rabbit IgG secondary antibodies (1:500–1:1000; Life Technologies, Waltham, MA, USA).

The following primary antibodies were used for immunoblotting: rabbit monoclonal anti-phospho-histone H2A.X (Ser139) (γH2AX) (1:500; 2577S, Cell Signaling Technology) and anti-phospho-Chk1 (Ser345) (1:1000; 133D3, Cell Signaling Technology) antibodies, rabbit polyclonal anti-phospho-KAP1 (Ser824) (1:2000; A300-767A, Bethyl Laboratories, Montgomery, TX, USA), anti-phospho-Chk2 (Thr68) (1:1000; 2661S, Cell Signaling Technology), anti-G3PDH (1:2000; 2275-PC-100, Trevigen), and anti-phospho-Src (Tyr416) (1:2000; #2101, Cell Signaling Technology) antibodies, mouse monoclonal anti-actin (1:2000; clone AC-40, Sigma-Aldrich), anti-Chk1 (1:800; G-4, Santa Cruz Biotechnology, Dallas, TX, USA), anti-Src (1:200; GD11, Millipore), anti-Chk2 (1:1000; clone DCS-273, Medical and Biological Laboratories, Nagoya, Japan), and anti-phosphotyrosine (1:400; clone 4G10, Merck Millipore) antibodies. Horseradish peroxidase-conjugated anti-mouse, anti-rabbit, anti-rat, and anti-goat IgG secondary antibodies (1:2000–1:4000; Santa Cruz Biotechnology), and anti-mouse IgG secondary antibody (1:4000; Cell Signaling) were used.

### 4.4. Immunofluorescence Microscopy

Immunofluorescence staining was performed as previously described [37,38,39,40]. For detection of γH2AX foci and pTyr, cells were washed with phosphate-buffered saline (PBS) containing Ca^2+^ and Mg^2+^ (PBS(+)) prewarmed at 37 °C and then fixed with PBS containing 4% paraformaldehyde and 20% methanol for 20 min at room temperature. The fixed cells were washed three times with PBS containing 10 mM sodium orthovanadate and 10 mM Hepes, and then permeabilized with methanol for 1 min at −30 °C. Then, the cells were blocked with PBS containing 0.1% saponin and 3% bovine serum albumin (BSA). Subsequently, cells were incubated with a primary and secondary antibody for 1 h each at room temperature. DNA was stained with 1 μM Hoechst 33342 for 1 h together with the secondary antibody. We observed fluorescence images under an IX-83 fluorescence microscope (Olympus, Tokyo, Japan) equipped with a 40 × 0.75 NA objective, a 60 × 1.42 NA oil-immersion objective, or a 100 × 1.40 NA oil-immersion objective (Olympus). The optical system for fluorescence observations included a U-FUNA cube (360–370 nm excitation, 420–460 nm emission) for observing Hoechst 33342 fluorescence, U-FBNA cube (470–495 nm excitation, 510–550 nm emission) for Alexa Fluor 488 fluorescence, and U-FRFP cube (535–555 nm excitation, 570–625 nm emission) for Alexa Fluor 555 fluorescence. Exposure time and lamp power were set and kept constant throughout each experiment to avoid overexposure of the fluorescence signal. Composite microscopic images were edited using ImageJ software (The National Institutes of Health (NIH), Bethesda, MD, USA) and Illustrator CC software (Adobe, San Jose, CA, USA).

### 4.5. Image Analysis

Image acquisition was performed by a high-content automated imaging system (Operetta, Perkin-Elmer, Waltham, MA, USA) with a 40× objective. Acquired images were analyzed using Harmony software (PerkinElmer). Tyrosine phosphorylation levels were evaluated by analyzing the integrated signal intensity per cell. To evaluate the level of phosphorylation of histone H2AX (γH2AX), the number of foci inside the nucleus was counted and the integrated foci area was determined in the nucleus of each individual cell. For this purpose, staining with Hoechst 33342 was used to identify the nuclei.

### 4.6. Western Blot Analysis

Cell lysates that were dissolved with an SDS-sample buffer were separated by SDS-PAGE and electrotransferred onto polyvinylidenedifluoride (PVDF) membranes (Pall Corporation, Port Washington, NY, USA). To examine the phosphorylation of proteins, cells were dissolved with an SDS-sample buffer containing the phosphatase inhibitors 20 mM β-glycerophosphate, 50 mM NaF, and 10 mM sodium orthovanadate. Blots were incubated with Blocking One (03953-95, Nakalai Tesque, Kyoto, Japan) on the rocking shaker for 30 min at room temperature, and sequentially probed with primary and secondary antibodies that were diluted with 0.1% Tween 20-conaining Tris-buffered saline for 1 h at room temperature or overnight at 4 °C. Chemiluminescence was detected with the image analyzer ChemiDoc XRSplus (Bio-Rad, Hercules, CA, USA) using Chemi Lumi-One L (#07880, Nacalai Tesque) or Clarity (170-5061, Bio-Rad) as the chemiluminescence substrate. The amount of protein was quantified by measuring the signal intensities of the bands using ImageJ software (NIH, USA).

## Abbreviations

ADR	Adriamycin
BLM	bleomycin
Dox	doxycycline
HR	homologous recombination
NHEJ	nonhomologous end joining
PBS	phosphate buffered saline
